# Supplementary material for: Deployable CRISPR-Cas13a diagnostic tools to detect and report Ebola and Lassa virus cases in real-time
Source: Nat Commun. 2020 Aug 17;11:4131. doi: 10.1038/s41467-020-17994-9 (PMC7431545; doi:10.1038/s41467-020-17994-9)
Supplement: Supplementary file 1 — Supplementary Information [file 41467_2020_17994_MOESM1_ESM.pdf]

Deployable CRISPR-Cas13a diagnostic tools to detect and report Ebola and Lassa virus cases in real-time

Supplementary Appendix

Table of Contents: Supplementary Figures and Table

Supplementary Figure 1. LASV SHERLOCK development, optimization and validation
Supplementary Figure 2. Field-deployable SHERLOCK protocol
Supplementary Figure 3. HandLens mobile app
Supplementary Figure 4. EBOV SHERLOCK development, optimization and validation

Supplementary Table 1. RPA primer sequences
Supplementary Table 2. crRNA spacer sequences
Supplementary Table 3. Virus-specific SHERLOCK assays
Supplementary Table 4. RT-qPCR primer sequences
Supplementary Table 5. RT-qPCR probe sequences
Supplementary Table 6. IRF results

Supplementary Figure 1. LASV SHERLOCK development, optimization and validation
Detection of a serial dilution of LASV synthetic DNA using (a-b) fluorescence based on mean of three technical replicates and (c-d) lateral flow readouts. Synthetic DNA was clade-specific, and concentrations ranged from 10^5 cp/uL-1 cp/µL. Error bars are 1 SD based on 3 technical replicates. (a and c) LASV-II was developed to identify clade II, and (b and d) LASV-IV was developed to identify clade IV.

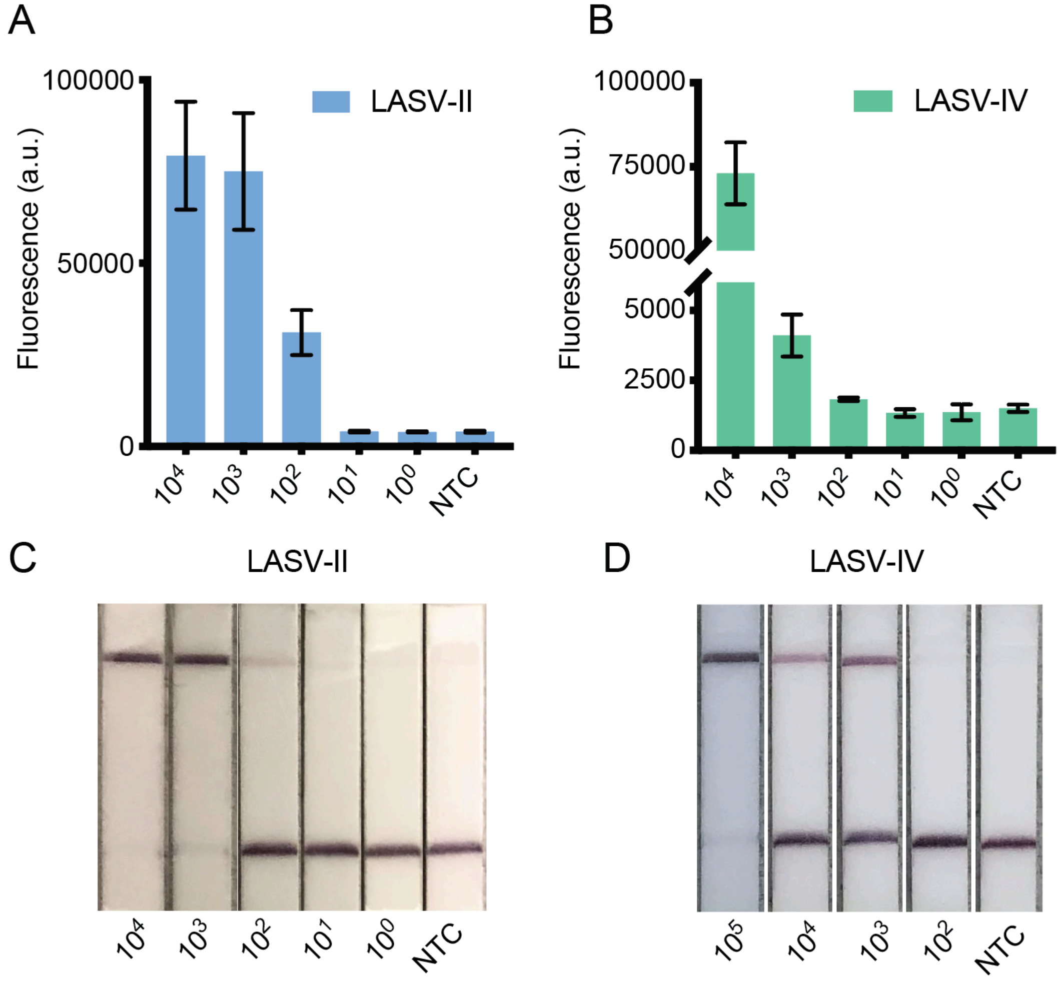

Supplementary Figure 2. Field-deployable SHERLOCK protocol

1-page, user-friendly SHERLOCK protocol used by collaborating institutions in Sierra Leone, Nigeria, and the USA. This field-deployable protocol simplifies SHERLOCK reactions and includes instructions for fluorescence or visual readout of the results.

SHERLOCK DETECTION PROTOCOL   Date:__________   Experiment name: ______________________

STEP 1 Viral amplification: RPA reaction

samples tested: ______
pellets needed: ______
(1 RPA pellet = 3.5 samples)

1. Make RPA mastermix:

Reagent	Per RPA pellet	Per __ pellet
Nuclease-free H20	3.7	
Primer mix (10 uM)	4.8	
Rehydration buffer	29.5	
Murine RNAse Inhibitor	5	

2. Use RPA mastermix to resuspend RPA pellets

RPA pellet	1	

3. Add MgAc to mastermix

MgAc	2.5	

4. Aliquot 9uL mastermix, 1uL sample into strip tubes or plate
5. Incubate on thermocycler at 41° C for 20 minutes

SHERLOCK Detection overview

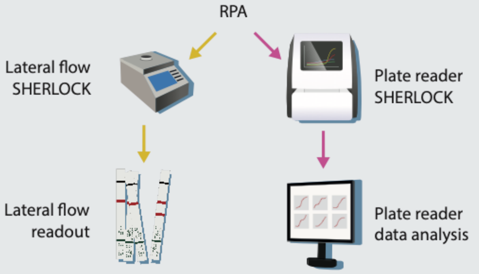

STEP 2 SHERLOCK : EITHER Lateral Flow reaction OR Plate reader reaction

SHERLOCK detection (for Lateral Flow strips)

1. Reconstitute Cas13a protein with 110.5 uL of SB. Reconstitute multiple proteins if necessary.
2. Make SHERLOCK Lateral Flow mastermix:

Reagent	Per 1 sample	Per ___ samples
Nuclease-free H20	15.17	
CB	2.50	
rNTPs (25 nM)	1.0	
RNase Inhibitor	1.25	
LF probe (100 uM)	0.25	
MgCl2 (1M)	0.23	
Reconstituted Cas13a	2.50	
T7 polymerase	0.75	
crRNA (guide)	0.11	
Total volume	23.76	

3. Aliquot 21.85 uL of mastermix per sample into plate
4. Aliquot 1.15 uL of RPA into plate
5. Seal plate, lightly vortex, and spin down
6. Incubate on thermocycler:

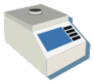

Cycler Conditions

Temp:	Time:
37° C	1H
4° C	hold

SHERLOCK detection (for plate reader)

1. Reconstitute Cas13a protein with 110.5 uL of SB. Reconstitute multiple proteins if necessary.
2. Make SHERLOCK plate reader mastermix:

Reagent	Per 1 sample	Per ___ samples
Nuclease-free H20	41.57	
CB	7.50	
rNTPs (25 nM)	3.00	
RNase Inhibitor	3.75	
Substrate (v2)	4.69	
Reconstituted Cas13a	7.50	
T7 polymerase	2.25	
MgCl2 (1M)	0.68	
crRNA (guide)	0.33	
Total volume	71.26	

2. Aliquot 65.55 uL of mastermix per sample into strip tube
3. Add 3.45 uL of RPA per sample into strip tube
4. Aliquot 20 uL from strip tube into 96 well plate in 3 replicates
5. Seal plate, lightly vortex, and spin down
6. Incubate on plate reader:

Profile Conditions
Temp: 37° C   Time: 1H

Measurement Conditions
Excitation: 485nm
Emission: 520 nm
Reading every 5 minutes

STEP 3 Result readout: EITHER Lateral Flow strips OR Plate reader analysis

Lateral flow readout

1. Add 80uL of Hybridetect Assay Buffer directly into each 23 uL reaction from Step 2.
2. Insert strips into reactions (arrows pointed up)
3. Incubate at room temperature for 5-15 minutes
4. Remove strips. Photograph with phone camera

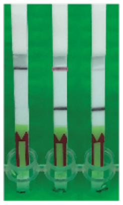

A B C

(A) Top fluorescent band = positive
(B) Faint top band with bottom band = positive
(Caused by lower target concentration within the tested sample)
(C) Bottom band only = negative

Plate reader data analysis

1. Export data from plate reader
2. Analyze data using the SHERLOCK Excel macro

Supplementary Figure 3. HandLens mobile app

(a) Receiver Operator Characteristic Curve (ROC) generated from running the reader algorithm on a total of 21 strips from 4 different EBOV dilution series. Of those, 17 are true positives. The AUC is 0.95, with a 95% CI of (0.88, 1.00). Using a positive classification threshold of 0.7, the algorithm exhibited the following performance indices: accuracy = 0.93, sensitivity = 0.91, and specificity = 1.00.

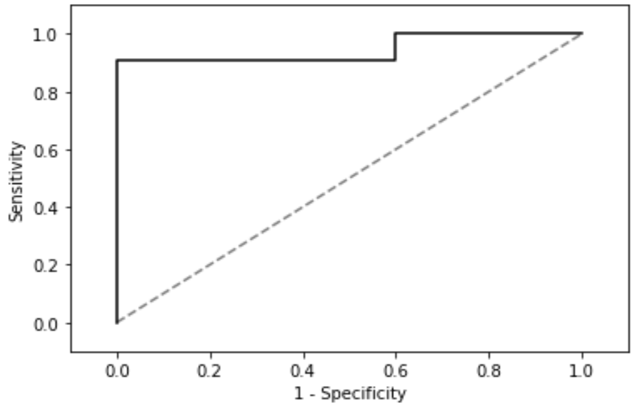

(b) The following images illustrate challenges in band detection. In the strips shown in the raw image data, the second from the right should be negative but is classified as positive with the default classification threshold of 0.5. In the CLAHE-filtered image, it is apparent that the enhancement creates a significant bump (visible in the second linearized signal plot from the top, pink line) that goes over the threshold (gray line). We currently handle these issues by increasing the classification threshold to a higher value (0.7). We plan to improve the image filtering algorithm to better handle these artifacts in the next version of the reader.

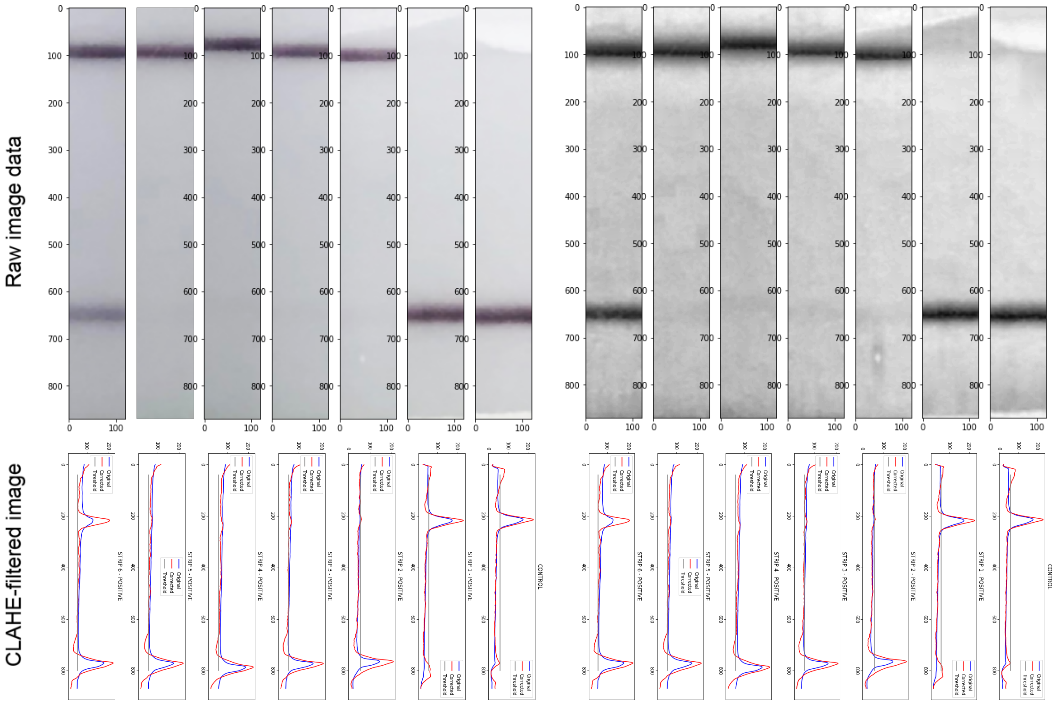

Supplementary Figure 4. EBOV SHERLOCK development, optimization and validation
(a) We developed numerous crRNAs targeting the L (EBOVA and EBOVD) and NP (EBOVB and EBOVC) genes. (b) Panel shows the fluorescence of SHERLOCK reactions run using each crRNA over a 3-hour time course with a fluorescent measurement taken every 5 minutes. Input for each reaction was synthetic cDNA at a concentration of 10^4 cp/µL (line) and NTC (triangle). crRNAs EBOVA and EBOVB reported the highest fluorescence compared to background. (c and d) We conducted LOD experiments on crRNAs EBOVA and EBOVB using fluorescent based in mean of three technical replicates (c) and lateral flow (d) readouts. Input was synthetic cDNA at a concentration of 10^4 cp/µL, and error bars are 1 SD based on 3 technical replicates.

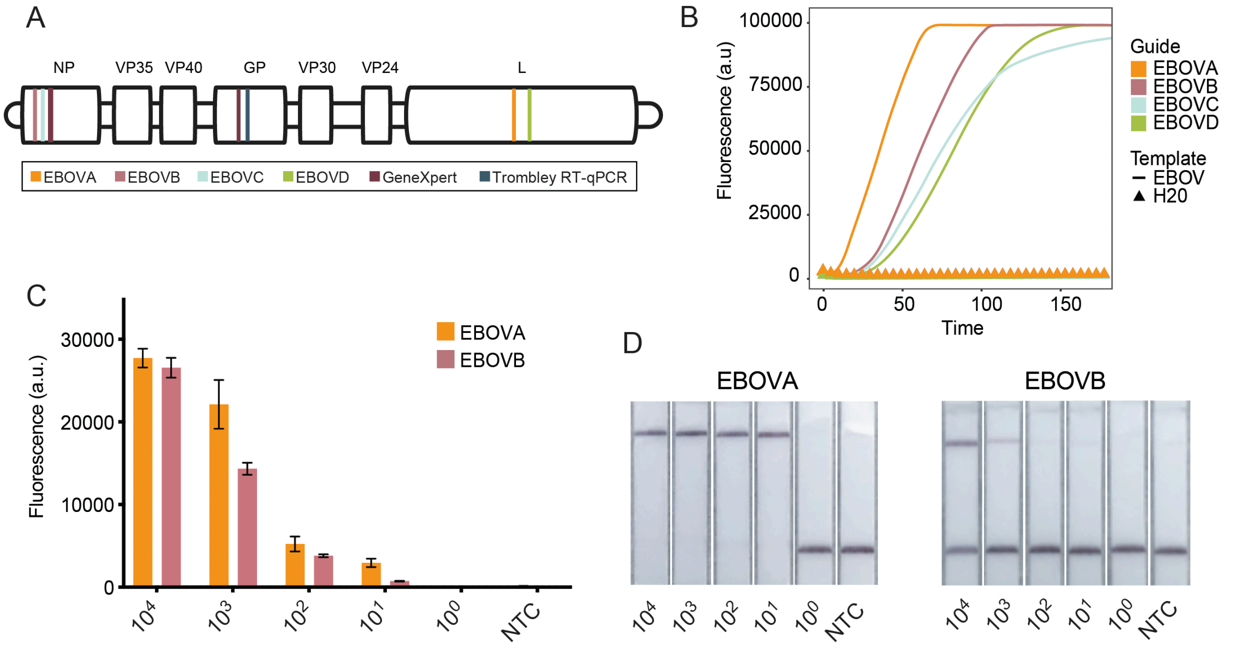

Supplementary Table 1. RPA primer sequences

RPA primer name	Sequence
EBOV-A_F	gaaatTAATACGACTCACTATAgggGACAGACTGAGGAARATAACATTGCAAAG
EBOV-A_R	CAATCATACATGGRAGTGTGGCTCCAATAA
EBOV-B_F	gaaatTAATACGACTCACTATAgggCAGTCAAGTAYTTGGAAGGGCACGGGTTC
EBOV-B_R	CTACTACCAATTTCGGAAGGAATAGACTTG
LASV-II_F	gaaatTAATACGACTCACTATAgggAAYCTYTCYGAYGCVCAYARRARGRAYCT
LASV-II_R	CRCCCCARGCCATYCTCATRAADGTYTG
LASV-IV_F	gaaatTAATACGACTCACTATAgggCATYGMATCYTTGAGRGTCAT
LASV-IV_R	AGGAATCCTTATGARAACATACTCTAYAA

Supplementary Table 2. crRNA spacer sequences

crRNA name	Spacer sequence
EBOVA	TTTAACCCAAATAACTTGCACAGTTGAT
EBOVB	AGAACACTTGCTGCCATGCCGGAAGAGG
LASV-IIA	CYYTRATGAGYATYATYTCAACYTTCCA
LASV-IIB	AATCARTATGARGCRATGAGYTGTGAYT
LASV-IIC	AYTTYAATCARTATGARGCRATGAGYTG
LASV-IVA	CTTCCTGTTATTGARGTYCTTGATGCAAT

Supplementary Table 3. Virus-specific SHERLOCK assays

Assay name	RPA primers used	crRNA used
EBOV	EBOV-A_F; EBOV-A_R	EBOVA
LASV-II	LASV-II_F; LASV-II_R	Equal mix of LASV-IIA and LASV-IIB
LASV-IV	LASV-IV_F; LASV-IV_R	LASV-IVA

Supplementary Table 4. RT-qPCR primer sequences

Primer name	Sequence	Reference
Nikisins_F	CCACCATYTTRTGCATRTGCCA	Nikisins *et al.* 2015
Nikisins_R	GCACATGTNTCHTAYAGYATGGAYCA	Nikisins *et al.* 2015
Broad_F	GATGCRGCYRAYCAYTGTG	This study
Broad_R	GARAACTGGCAGTGATCTTCC	This study

Supplementary Table 5. RT-qPCR probe sequences

Probe name	Sequence	Reference
Nikisins_P	FAM-AARTggggYCCDATgATgTgYCCWTT-BBQ	Nikisins *et al.* 2015
Broad_P	/56-FAM/TT YAT GAG G/ZEN/A TGG CTT GGG GTG G/3IABkFQ/	This study

Supplementary Table 6. IRF results

§Titers were determined by plaque assay. Each PFU/mL titer is accurate to ± a half-log. ∅Not Detected – No plaques formed. ßFewer than 10 plaques per well. The lower limit of detection for the plaque assay is 100 PFU/mL ± a half-log.

**Blood**				
Anticipated Titer	§Titer (PFU/mL)	Lateral Flow Results (95°C)	Lateral Flow Results (70°C)	GeneXpert Results (ct-value)
10^0	∅Not Detected	Negative	Negative	NP detected-38.1<br>GP NOT detected
10^1	∅Not Detected	Negative	Negative	GP detected-42.1<br>NP detected-36.1
10^2	4.44E+02	Negative	Negative	GP detected-36.1<br>NP detected-31.6
10^3	1.14E+04	Negative	Negative	GP detected-33.2<br>NP detected-28.4
10^4	1.07E+05	Positive	Positive	GP detected-29.5<br>NP detected-24.9
10^5	7.44E+05	Positive	Positive	GP detected-26.4<br>NP detected -21.5
**Urine**				
Anticipated Titer	§Titer (PFU/mL)	Lateral Flow Results (95°C)	Lateral Flow Results (70°C)	GeneXpert Results (ct-value)
10^0	ßBelow LLOD	Negative	Negative	NP detected-36.9<br>GP NOT detected

10^1	1.06E+02	Negative	Negative	GP detected-40.8<br>NP detected-34.1
10^2	1.10E+03	Negative	Negative	GP detected-35.9<br>NP detected-31.4
10^3	1.08E+04	Negative	Negative	GP detected-33.1<br>NP detected-28.2
10^4	1.17E+05	Positive	Positive	GP detected-31.5<br>NP detected-26.2
10^5	1.08E+06	Positive	Positive	GP detected-26.5<br>NP detected -21.4
**Saliva**				
Anticipated Titer	§Titer (PFU/mL)	Lateral Flow Results (95°C)	Lateral Flow Results (70°C)	GeneXpert Results (ct-value)
10^0	øNot Detected	Negative	Negative	NP detected-42.1<br>GP NOT detected
10^1	4.89E+01	Negative	Negative	GP detected-41.0<br>NP detected-37.4
10^2	1.06E+03	Negative	Negative	GP detected-37.4<br>NP detected-33.1
10^3	9.44E+03	Positive	Positive	GP detected-35.3<br>NP detected-30.7
10^4	1.26E+05	Positive	Positive	GP detected-32.2<br>NP detected-27.4
10^5	1.50E+06	Positive	Positive	GP detected-25.5<br>NP detected -20.2
